# Supplementary material for: Development of a Polymeric Pharmacological Nanocarrier System as a Potential Therapy for Spinocerebellar Ataxia Type 7
Source: Cells. 2023 Nov 30;12(23):2735. doi: 10.3390/cells12232735 (PMC10706302; doi:10.3390/cells12232735)
Supplement: Supplementary file 1 [file cells-12-02735-s001.zip › cells-2674953-supplementary.pdf]

Supplementary Table S1

Supplementary Table S1.

A) DSC of nanoparticulate formulations and raw materials.

**Sample**	**T1 (°C)**	**T2 (°C)**	**T3 (°C)**
PLGA	51	246	--
PVA	70.9	195	--
Plx188	56	--	--
Rapamycin	186	197	
PLGA NPs	54	156	166
Rap-PLGA NPs	53	155	165

B) TGA of nanoparticulate formulations and excipients.

**Sample**	**T1 (°C)**	**T2 (°C)**
PLGA	195-349	--
PVA	257-365	380-468
Plx188	250-363	--
Rapamycin	196-460	220-250
PLGA NPs	216-329	--
Rap-PLGA NPs	216-326	--

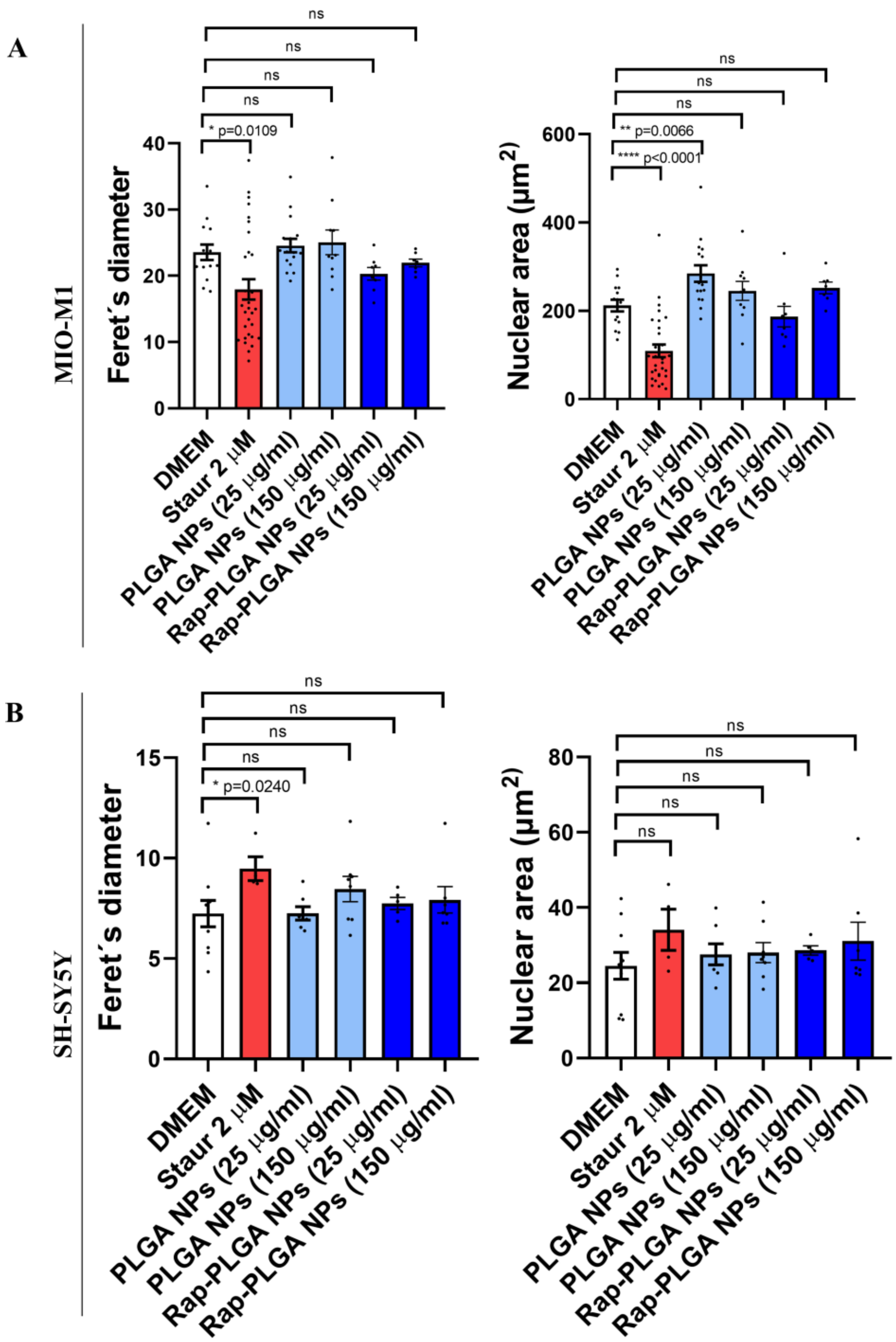

Figure S1. Treatment in MIO-M1 and SH-SY5Y cells with PLGA NPs or Rap-PLGA NPs has no effect on cellular or nuclear morphology. Cells were incubated for 72 h with 25 µg/mL or 150 µg/mL of PLGA NPs or Rap-PLGA NPs prior to be subjected to rhodamine-conjugated phalloidin (Ph) or DAPI staining, to decorate the actin-based cytoskeleton and nuclei respectively. Cells were subjected to Confocal Laser Scanning Microscopy analysis (Figure 6) and further morphometric analyses using Image J, to obtain Feret's diameter and cellular and nuclear area measurements. Significant differences were determined by Mann-Whitney U test (n > 4 per condition) from three independent experiments; A) MIO-M1; B) SH-SY5Y. Bars indicate mean + SEM

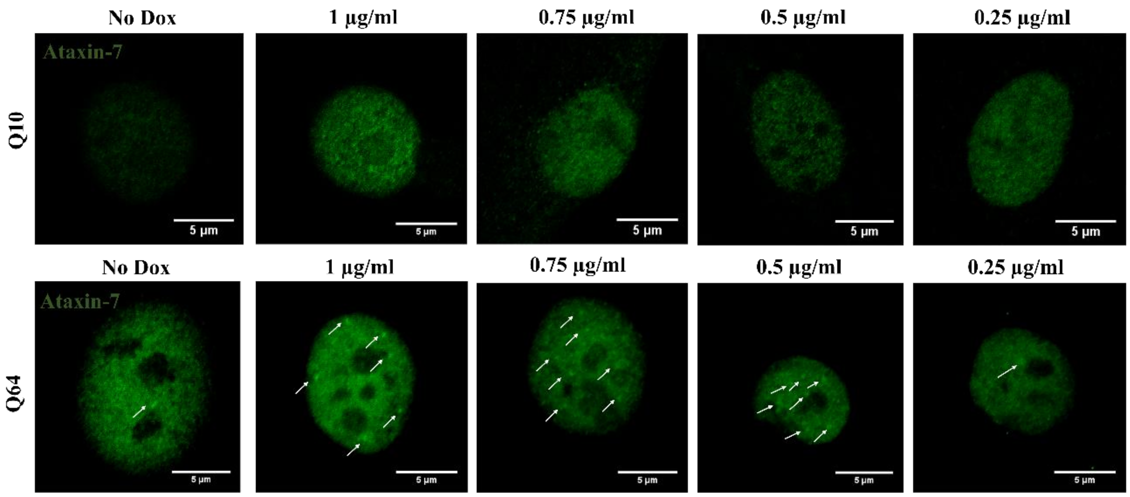

Figure S2. Calibration curve of doxycycline in the inducible model with Q10 and Q64. The figure displays the calibration curve for doxycycline using the Q10 and Q64 cell lines. Each cell line is depicted with its respective control (No Doxycycline – No Dox) and varying concentrations of doxycycline (1 µg/mL, 0.75 µg/mL, 0.5 µg/mL, and 0.25 µg/mL). Cells preparations were then marked with anti-ataxin-7 antibody and subjected to Confocal Laser Scanning Microscopy analysis.

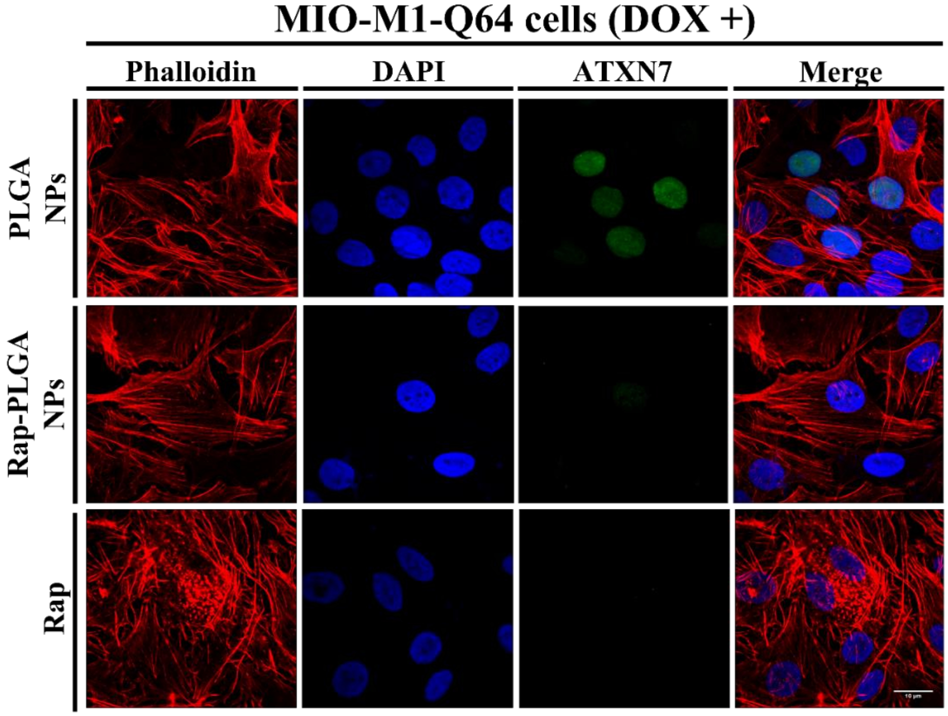

Figure S3. Analysis of MIO-M1-Q64 cell morphology after treatment with NPs and rapamycin. Induction of the model with Dox during 24 h and treated with PLGA NPs, Rap-PLGA NPs or Rap for 24 h prior to be stained with rhodamine-conjugated phalloidin, DAPI, and ataxin-7 to decorate the actin-base cytoskeleton, nuclei, and Atxn7 expression, respectively. Cells preparations were then subjected to Confocal Laser Scanning Microscopy analysis.
